# scdNet: a computational tool for single-cell differential network analysis

**DOI:** 10.1186/s12918-018-0652-0

**Published:** 2018-12-21

**Authors:** Yu-Chiao Chiu, Tzu-Hung Hsiao, Li-Ju Wang, Yidong Chen, Yu-Hsuan Joni Shao

**Affiliations:** 1 Greehey Children’s Cancer Research Institute, University of Texas Health Science Center at San Antonio, San Antonio, TX 78229 USA; 2 Department of Medical Research, Taichung Veterans General Hospital, Taichung, 40705 Taiwan; 3 Department of Epidemiology and Biostatistics, University of Texas Health Science Center at San Antonio, San Antonio, TX 78229 USA; 4 Graduate Institute of Biomedical Informatics, College of Medical Science and Technology, Taipei Medical University, Taipei, 10675 Taiwan

**Keywords:** Differential network analysis, Gene regulatory networks, Single-cell RNA-Seq

## Abstract

**Background:**

Single-cell RNA sequencing (scRNA-Seq) is an emerging technology that has revolutionized the research of the tumor heterogeneity. However, the highly sparse data matrices generated by the technology have posed an obstacle to the analysis of differential gene regulatory networks.

**Results:**

Addressing the challenges, this study presents, as far as we know, the first bioinformatics tool for scRNA-Seq-based differential network analysis (scdNet). The tool features a sample size adjustment of gene-gene correlation, comparison of inter-state correlations, and construction of differential networks. A simulation analysis demonstrated the power of scdNet in the analyses of sparse scRNA-Seq data matrices, with low requirement on the sample size, high computation efficiency, and tolerance of sequencing noises. Applying the tool to analyze two datasets of single circulating tumor cells (CTCs) of prostate cancer and early mouse embryos, our data demonstrated that differential gene regulation plays crucial roles in anti-androgen resistance and early embryonic development.

**Conclusions:**

Overall, the tool is widely applicable to datasets generated by the emerging technology to bring biological insights into tumor heterogeneity and other studies. MATLAB implementation of scdNet is available at https://github.com/ChenLabGCCRI/scdNet.

## Background

Single-cell sequencing is a developing technology that enables a close look into the heterogeneity and clonal evolution of cancer cells. While many methods have been designed to analyze single-cell DNA-Seq data [1], the analysis of scRNA-Seq data remains challenging due to high sparsity that prevents direct applications of methods originally developed for microarray and bulk RNA sequencing. On the other hand, the analysis of gene regulatory networks is a widely used approach to realize the signaling and interactions among genes. Recent studies have successfully applied correlation onto the inference of gene regulatory networks by using scRNA-Seq data [2, 3]. However, realizing that tumor cells are highly heterogeneous, network topologies may be massively changed between cells of different cellular states [4]. Recently, a computational method was proposed to investigate the change in mean absolute distances of a gene to others [5]. However, the computational method for studying individual gene pairs of which regulatory strengths alter between conditions was only carried out in the bulk RNA sequencing data [6–8].

Addressing this research need, here we developed a comprehensive bioinformatics tool for single cell-based differential network analysis, namely scdNet. It features two main functions: i) gene correlation analysis out of highly sparse data matrices and ii) differential network analysis between cellular states. Performance of scdNet was tested by simulated datasets. We further applied the tool to scRNA-Seq datasets of CTCs and early-stage mouse embryos for differential networks associated with anti-androgen resistance of prostate cancer and early embryonic development.

## Methods

### Transformation of intra-state gene-gene correlation

Suppose a scRNA-Seq dataset ***E***_***G*** × ***K***_ = {*e*(*g*, *k*)} contains read counts normalized by DESeq2 [9] of the *g*-th genes in the *k*-th single cells (*g* ∈ [1, *G*] and *k* ∈ [1, *K*]). We *z*-transformed ***E*** with respect to genes to eliminate biases: ${\boldsymbol{E}}_{\boldsymbol{G}\times \boldsymbol{K}}^{\boldsymbol{z}}=\left\{\frac{e\left(g,k\right)-{\mu}_g}{\sigma_g}\right\}$, where *μ*_*g*_ and *σ*_*g*_ are mean and standard deviation values of gene *g* across *K* cells. For the sparsity of scRNA-Seq data, we adopted a sample size adjustment and comparison of Pearson correlation coefficients among states. Within a cellular state *n* (*n* ∈ {0, 1}), gene-gene correlation coefficients were computed into the correlation matrix ${\boldsymbol{C}}_{\boldsymbol{G}\times \boldsymbol{G}}^{\boldsymbol{n}}$:

1 $$ {\boldsymbol{C}}^{\boldsymbol{n}}\left(i,j\right)=\rho \left(\boldsymbol{E}\left(i,{\boldsymbol{x}}_{\boldsymbol{i},\boldsymbol{j}}\right),\boldsymbol{E}\left(j,{\boldsymbol{x}}_{\boldsymbol{i},\boldsymbol{j}}\right)\right), $$

where (*i*, *j*) ∈ [1, *G*] × [1, *G*] and ***x***_***i***, ***j***_ = {*s*| ***E***(*i*, *s*) ≠ 0} ∩ {*t*| ***E***(*j*, *t*) ≠ 0} for samples of the state. Number of samples used to calculate the correlation was stored in the sample size matrix ${\boldsymbol{S}}_{\boldsymbol{G}\times \boldsymbol{G}}^{\boldsymbol{n}}$:

2 $$ {\boldsymbol{S}}^{\boldsymbol{n}}\left(i,j\right)=\parallel {\boldsymbol{x}}_{\boldsymbol{i},\boldsymbol{j}}\parallel . $$

We applied the Fisher transformation $\mathcal{F}$, as described in our previous studies [6–8], to convert the correlation coefficients into a sample size-free domain and termed as the interaction matrix ${\boldsymbol{I}}_{\boldsymbol{G}\times \boldsymbol{G}}^{\boldsymbol{n}}$:

3 $$ {\boldsymbol{I}}^{\boldsymbol{n}}\left(i,j\right)=\mathcal{F}\left({\boldsymbol{C}}^{\boldsymbol{n}}\left(i,j\right),{\boldsymbol{S}}^{\boldsymbol{n}}\left(i,j\right)\right)=\frac{\sqrt{{\boldsymbol{S}}^{\boldsymbol{n}}\left(i,j\right)-3}}{2}\ln \frac{1+{\boldsymbol{C}}^{\boldsymbol{n}}\left(i,j\right)}{1-{\boldsymbol{C}}^{\boldsymbol{n}}\left(i,j\right)}. $$

Elements of the interaction matrix followed the standard normal distribution.

### Inter-state differential network analysis

We analyzed the changes of interaction strengths of each gene-gene pair, say *i* and *j*, between two cellular states:

4 $$ \mathbf{\Delta }\boldsymbol{I}\left(i,j\right)=\left|{\boldsymbol{I}}^{\mathbf{1}}\left(i,j\right)\right|-\left|{\boldsymbol{I}}^{\mathbf{0}}\left(i,j\right)\right| $$

and tested for statistical significance by the cumulative distribution function (CDF) [8]:

5 $$ F\left(\mathbf{\Delta }\boldsymbol{I}\left(i,j\right)\right)=\frac{1}{2}+\mathit{\operatorname{erf}}\left(\frac{\mathbf{\Delta }\boldsymbol{I}\left(i,j\right)}{2}\right)-\frac{1}{2}\mathit{\operatorname{sgn}}\left(\mathbf{\Delta }\boldsymbol{I}\left(i,j\right)\right)\bullet {\left[\mathit{\operatorname{erf}}\left(\frac{\mathbf{\Delta }\boldsymbol{I}\left(i,j\right)}{2}\right)\right]}^2 $$,

where *erf*() and *sgn*() are the Gauss error function and sign function, respectively. Given the exact CDF of the changes in the interaction values, *P*-values are directly assessed and time-consuming permutation or simulation tests can be avoided. Gene pairs with significant changes were defined as differential pairs and merged into a differential network.

### Visualization and functional annotation analysis of gene regulatory networks

Networks of identified genes and their dynamic interactions across cellular states were visualized by the Cytoscape software (version 3.5.1) [10], with nodes and edges denoting genes and differential gene regulations, respectively. To investigate the functional relevance of cellular state-modulated differential networks, top hub genes of the network were analyzed for the associations with Gene Ontology (GO) terms of molecular functions, cellular components, and biological processes by the Database for Annotation, Visualization and Integrated Discovery (DAVID) [11].

## Results

### Model overview

**Fig. 1:**
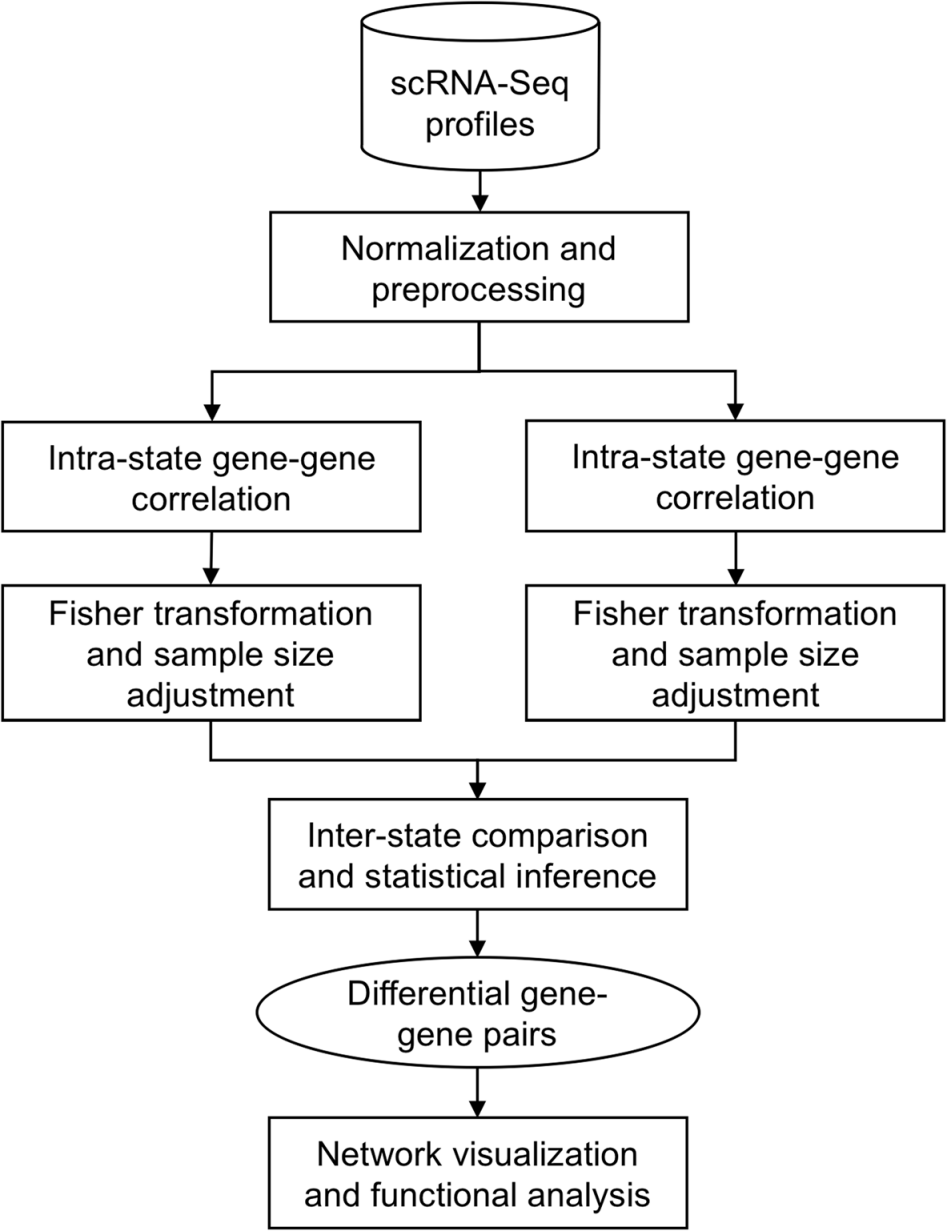
Flowchart of the proposed method. The method is designed to analyze differential gene regulatory networks from scRNA-Seq data. It features two functions: i) measurement of sample size corrected gene-gene correlation for each state to handle the sparse data matrices and ii) statistical inference of the changes in correlation across cellular states. The identified differential gene-gene pairs were subject to network and functional annotation analyses

scdNet is devised to analyze differential gene regulatory networks associated with cellular states at the single cell level. Fig. 1 shows the flowchart of the tool. Briefly, scRNA-Seq data were preprocessed and normalized to eliminate inter-cell biases, and non-informative genes with a coefficient of variation < 0.25 in either state were eleminated. Within each group of cells, gene-gene correlation coefficients were calculated with an exclusion of zeros, and transformed to a sample size independent domain by the Fisher transformation to eliminate sample size related biases. Normalized correlation coefficients were compared between groups of cells and the changes in correlation were statistically tested in the Fisher domain. Significantly changed gene-gene pairs were merged into a differential network. Visualization and functional annotation analyses were performed to realize the biological relevance of such dynamic network. MATLAB implementation of scdNet is available at https://github.com/ChenLabGCCRI/scdNet.

### Assessment of model performace – simulation design

We simulated scRNA-Seq datasets to test the performance of the proposed method in analyzing gene-gene correlations out of highly sparse scRNA-Seq data. Since we *z*-transformed the sequencing data, the synthetic datasets were generated by randomly sampling the standard normal distribution. We note that the performance of the comparison of interaction matrices (Eqs. 4–5) has been described in our previous papers [7, 8]. Thus, the simulation was simply focused on the comparison of intra-group correlations against zero (uncorrelated). In each simulation scenario, 10,000 gene pairs were generated, of which 20% were defined as correlated (covariance=*θ*), and the remaining 80% as independent (covariance = 0). Four parameters were tuned in the simulation: i) covariance of correlated gene pairs (*θ*, 0, 0.3, 0.7, and 1.0), ii) relative power of Gaussian noises added to the original signals to mimic sequencing errors (*ε*, 0, 0.05, 0.1, 0.25, 0.50, 0.75, 0.9, and 1.0), iii) number of single cells (*K*, 10, 20, 50,100, and 200), and iv) proportions of low-signal elements to be eliminated (representing zeroes of scRNA-Seq) (*τ*, 0, 0.25, 0.50, 0.75. 0.9, and 0.95). Yielded Fisher-transformed scores were compared to zero; gene pairs with Bonferroni-adjusted *P*-value< 0.05 were called as significant. Performance was evaluated by accuracy, sensitivity, specificity, and time consumption. The simulation processes were performed on MATLAB.

### Assessment of model performace by four parameters

#### Gene-gene Covariance (θ)

**Fig. 2:**
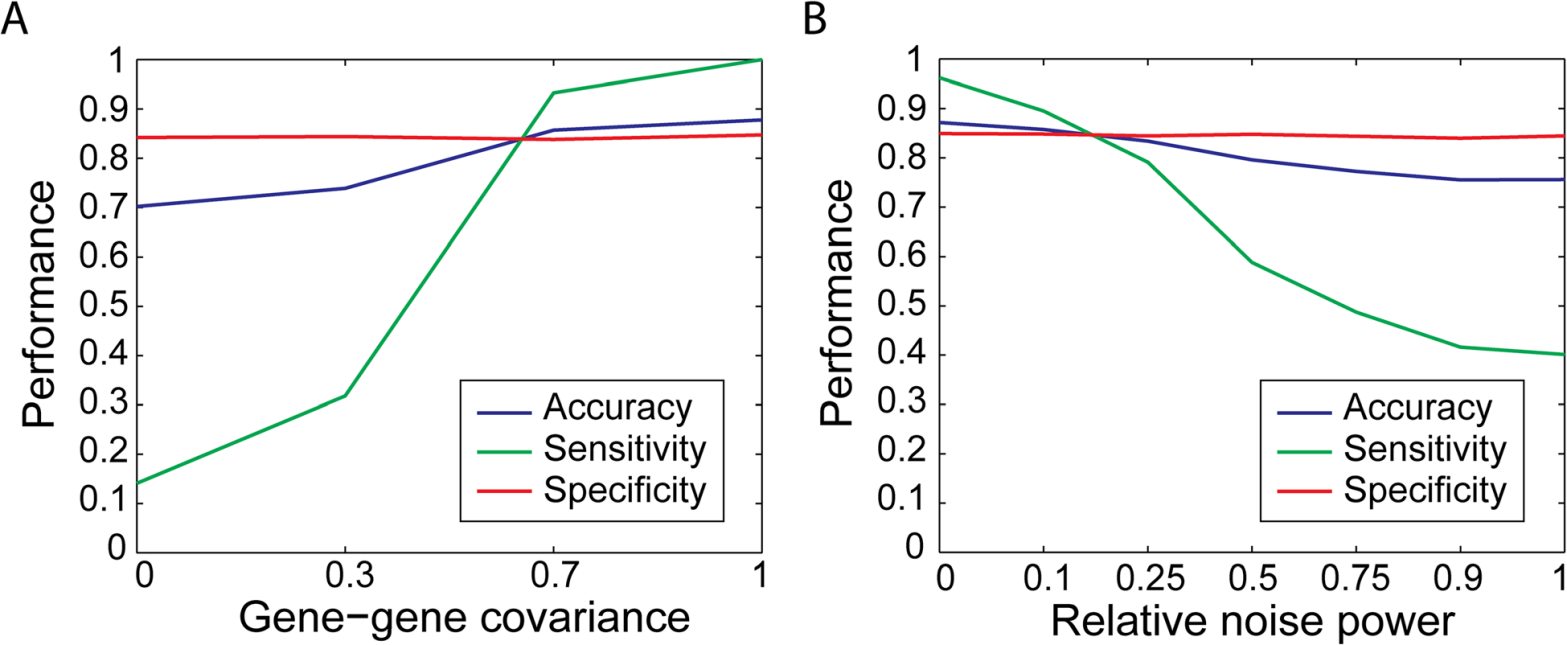
Simulation analysis for performance assessment with respect to gene-gene covariance and relative power of noise. **a** Performance of the tool on a dataset composed of 20% of correlated samples with a covariance ranging from 0 to 1 and 80% of uncorrelated samples. **b** Performance of the tool when Gaussian noises of different power relative to the original signals were added

With *ε*, *K*, and *τ* set at 0.05, 50, 0.25, respectively, we tested the performance of scdNet across different gene-gene covariance *θ*. As shown in Fig. 2a, the increase in *θ* greatly rose sensitivity from to 0.14 to 1.00 (*θ*=0 and 1.0) and slightly increased accuracy from 0.70 to 0.88. On the other hand, specificity seemed to be independent of *θ* (range, 0.84–0.85).

#### Relative Power of Noises (ε)

We then tested the effect of sequencing noises. Simulation settings were identical as described above, while *θ* was fixed at 0.7. At a general level of sequencing errors (*ε*=0.1), scdNet achieved high performance (accuracy, sensitivity, and specificity = 0.86, 0.90, and 0.85). While accuracy and specificity were not sensitive to *ε*, the sensitivity dropped to 0.40 when the noise was extremely high (Fig. 2b).

#### Number of Single Cells (K) and Proportion of Low-signal Elements (τ)

Due to limitations in budget and specimens, scRNA-Seq data are typically of limited sample sizes. To realize the effects of sample size and sparsity of scRNA-Seq data, we jointly analyzed the two factors. As shown in Table 1, the proposed tool achieved generally favorable performance in regardless of settings of *K* and *τ* when either group of cells had 10 or more non-zero values.

**Table 1:** Simulation analysis on the number of single cells and proportions of low-signal elements

Performance	Number of Cells (*K*)	Proportion of Low-signal Elements (*τ*)					
		0	0.25	0.50	0.75	0.90	0.95
Accuracy	10	0.84	0.77	0.81	–	–	–
	20	0.87	0.81	0.78	0.81	–	–
	50	0.88	0.87	0.81	0.83	0.80	0.80
	100	0.87	0.87	0.85	0.79	0.83	0.80
	200	0.87	0.88	0.87	0.81	0.84	0.82
Sensitivity	10	0.82	0.36	0.11	–	–	–
	20	0.98	0.65	0.35	0.03	–	–
	50	1.00	0.94	0.64	0.27	0.01	–
	100	1.00	1.00	0.86	0.45	0.14	0.00
	200	1.00	1.00	0.98	0.64	0.25	0.08
Specificity	10	0.85	0.87	0.99	–	–	–
	20	0.84	0.85	0.88	1.00	–	–
	50	0.85	0.85	0.85	0.97	1.00	–
	100	0.84	0.84	0.84	0.87	1.00	1.00
	200	0.84	0.85	0.84	0.86	0.98	1.00

#### Time Consumption

Facilitated by the exact probability function (Eq. 5), scdNet is of remarkably high computation efficiency. An inference of 10,000 gene pairs described above took an average of 1.3 s (std., 0.5) on a personal computer.

### Application to a prostate cancer dataset of CTCs

**Fig. 3:**
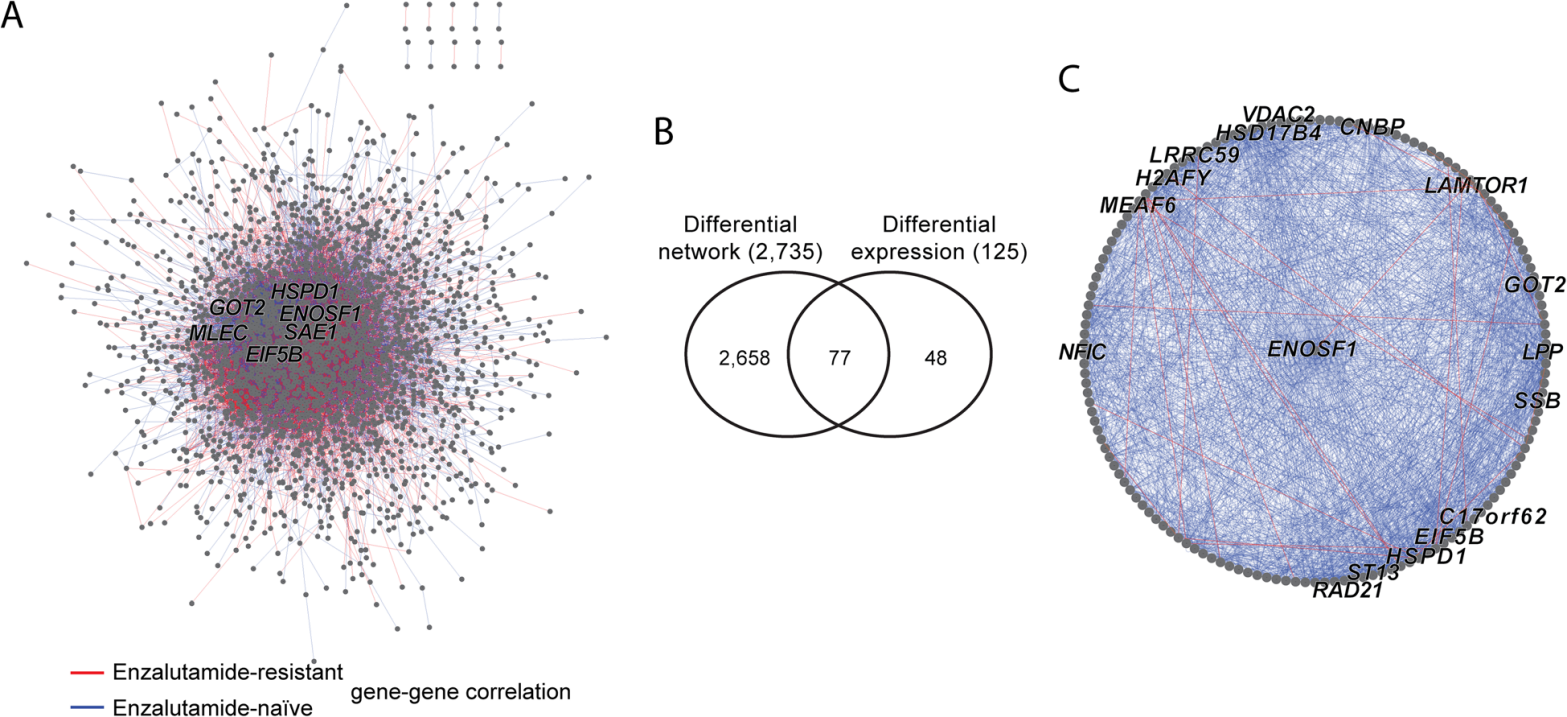
Differential gene networks associated with anit-androgen resistance of prostate cancer. **a** Differential gene regulatory network. The network was constructed by merging differential gene pairs between enzalutamide-resistant or -naïve CTCs, with nodes and edges representing genes and differential correlations, respectively. Top hub genes are labeled with gene symbols. **b** Venn diagram of genes involved in the differential network and those differentially expressed between two groups of cells. **c** Subnetwork of the top hub gene *ENOSF1*

We then applied scdNet to a real dataset derived from prostate CTCs. CTCs is an emerging technology of liquid biopsies that allows minimally invasive assessment and prediction of metastasis and treatment outcomes of advanced prostate cancer [12, 13]. Here we studied on the resistance of enzalutamide, a second-generation anti-androgen medication which is used in the treatment of prostate cancer [14, 15] while its resistance mechanisms remain vague. Thus, we utilized a public dataset of 169 scRNA-Seq of prostate CTCs (Gene Expression Omnibus accession number: GSE67980) [16]. We normalized raw read counts by DESeq2. The dataset was found very sparse. Among 21,696 unique genes, each cell carried an average of ~ 76.4% genes (16,573, std., 2293) with no sequencing reads. We analyzed 77 samples isolated from 13 patients, of which 41 progressed on enzalutamide (hereafter referred to as the enzalutamide-resistant group) and 36 were enzalutamide-naïve. Out of ~ 4.7 million transcriptome-wide gene pairs, we set a stringent criterion to identify the most significant subset of pairs that exhibited significant changes between the two groups (Bonferroni adjusted *P* < 1 × 10^− 5^). In total, 6023 and 10,670 pairs of genes were correlated with each other specifically in enzalutamide-resistant and -naïve groups, respectively, involving 2735 genes. These gene pairs formed a highly intertwined network (Fig. 3a); on average, each gene was connected to 12.2 partner genes. We note that only 2.8% of these genes were differentially expressed (with *t*-test *P* < 0.05) between the two groups of cells, confirming that our analysis was not biased by differential expression. (Fig. 3b). Table 2 tabulates the top 10 hub genes of the network. *ENOSF1*, a mitochondria enzyme that have been shown as a serum biomarker for gastric cancer [17], was the top hub with 150 differential pairs (Fig. 3c). Consistently, functional annotation analysis of the top 100 hub genes in the network also highlighted the association between mitochondria-related biological functions and anti-androgen resistance (Table 3). Thus, our data indicate differential gene regulatory networks, at least partially, govern mitochondria functions and play an essential role in anti-androgen resistance of prostate cancer.

**Table 2:** Top hub genes in the differential gene network between enzalutamide-resistant and -naïve CTCs

Gene symbol	Gene name	Num. differential pairs
*ENOSF1*	Enolase superfamily member 1	150
*EIF5B*	Eukaryotic translation initiation factor 5B	122
*HSPD1*	Heat shock 60 kDa protein 1 (chaperonin)	107
*SAE1*	SUMO1 activating enzyme subunit 1	106
*GOT2*	Glutamic-oxaloacetic transaminase 2, mitochondrial (aspartate aminotransferase 2)	102
*MLEC*	Malectin	102
*TXNIP*	Thioredoxin interacting protein	99
*LPP*	LIM domain containing preferred translocation partner in lipoma	98
*RAD21*	RAD21 homolog (S. pombe)	97
*EIF6*	Eukaryotic translation initiation factor 6	95

**Table 3:** Gene Ontology terms associated with top 100 hub genes of the enzalutamide resistance-modulated differential network

Category	Term	Gene count	*P*-value
Annotation Cluster 1 (Enrichment Score: 3.43)			
CC	GO:0044429~mitochondrial part	15	5.6 × 10^−6^
CC	GO:0005739~mitochondrion	19	2.9 × 10^−5^
CC	GO:0031980~mitochondrial lumen	8	3.1 × 10^−4^
Annotation Cluster 2 (Enrichment Score: 3.17)			
CC	GO:0044429~mitochondrial part	15	5.6 × 10^−6^
CC	GO:0005740~mitochondrial envelope	12	2.4 × 10^−5^
CC	GO:0005739~mitochondrion	19	2.9 × 10^−5^
Annotation Cluster 3 (Enrichment Score: 2.38)			
CC	GO:0031974~membrane-enclosed lumen	26	1.9 × 10^−5^
CC	GO:0070013~intracellular organelle lumen	24	9.0 × 10^−5^
CC	GO:0043233~organelle lumen	24	1.3 × 10^−4^
Annotation Cluster 4 (Enrichment Score: 1.90)			
MF	GO:0003743~translation initiation factor activity	5	4.6 × 10^−4^
MF	GO:0008135~translation factor activity, nucleic acid binding	5	2.7 × 10^−3^
BP	GO:0006412~translation	8	3.2 × 10^−3^
Annotation Cluster 5 (Enrichment Score: 1.74)			
BP	GO:0016071~mRNA metabolic process	8	5.8 × 10^−3^
BP	GO:0006397~mRNA processing	7	1.1 × 10^−2^
BP	GO:0000375~RNA splicing, via transesterification reactions	5	1.2 × 10^− 2^

Each cluster is represented by the top three terms

Abbreviations: *BP* biological process, *CC* cellular component, *MF* molecular function

### Application to a dataset of early mouse embryos

**Fig. 4:**
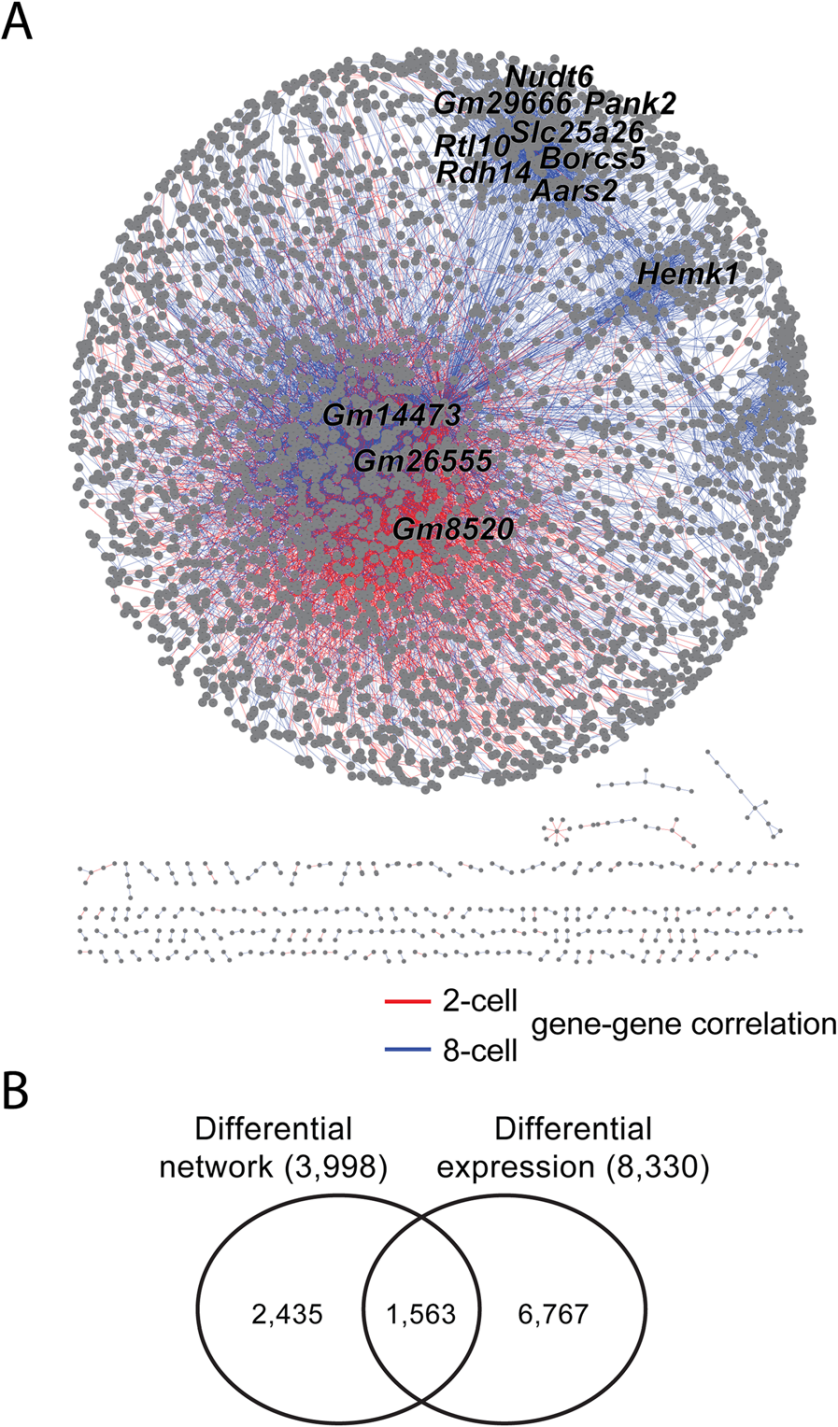
Differential gene networks associated with early development of mouse embryos. **a** Differential gene regulatory network of differential gene pairs identified by comparing the 2-cell and 8-cell stages of mouse embryonic development. Top hub genes are labeled with gene symbols. **b** Venn diagram of genes involved in the differential network and those differentially expressed between two groups of cells

We also applied scdNet to study the differential network associated with early development of mouse embryos. We preprocessed scRNA-Seq data of 32 single cells at the 8-cell stage and 16 cells at the 2-cell stage (ArrayExpress accession number E-MTAB-3321 [18]) as described in the CTC study. The data were also highly sparse (68.6% sparsity). Using identical criteria, we identified 11,245 gene regulatory pairs specifically shown in 2-cell (2915 pairs) and 8-cell (8330) embryos composed of 3998 genes (Fig. 4a). Again, the proportion of these genes to be differentially expressed was lower than expectation (39.1% compared to an expectation of 49.0%; Fig. 4b). The top 100 hubs genes were significantly associated with cell division and differentiation-related functions, such as cytoskeleton and chromatin assembly, and the ribosome and mitochondria, which are known to regulate early development of mouse embryos [19, 20] (Table 4).

**Table 4:** Gene Ontology terms associated with top 100 hub genes of the early embryonic development-modulated differential network

Category	Term	Gene count	*P*-value
Annotation Cluster 1 (Enrichment Score: 2.47)			
CC	GO:0043228~non-membrane-bounded organelle	14	4.9 × 10^−4^
CC	GO:0043232~intracellular non-membrane-bounded organelle	14	4.9 × 10^−4^
CC	GO:0005856~cytoskeleton	6	0.15
Annotation Cluster 2 (Enrichment Score: 1.62)			
MF	GO:0030554~adenyl nucleotide binding	10	1.6 × 10^−2^
MF	GO:0001883~purine nucleoside binding	10	1.6 × 10^−2^
MF	GO:0001882~nucleoside binding	10	1.7 × 10^−2^
Annotation Cluster 3 (Enrichment Score: 1.40)			
CC	GO:0005840~ribosome	4	1.3 × 10^−2^
BP	GO:0006412~translation	5	1.3 × 10^−2^
CC	GO:0030529~ribonucleoprotein complex	5	2.9 × 10^−2^
Annotation Cluster 4 (Enrichment Score: 0.79)			
BP	GO:0006333~chromatin assembly or disassembly	3	3.9 × 10^−2^
CC	GO:0000785~chromatin	3	6.6 × 10^−2^
CC	GO:0044427~chromosomal part	3	0.20
Annotation Cluster 5 (Enrichment Score: 0.64)			
CC	GO:0044429~mitochondrial part	4	0.15
CC	GO:0031967~organelle envelope	4	0.16
CC	GO:0031975~envelope	4	0.16

Each cluster is represented by the top three terms

Abbreviations: *BP* biological process, *CC* cellular component, *MF* molecular function

## Discussion

Recent advances in scRNA-Seq technology have revolutionized the investigation of tumor heterogeneity [21–23] and construction of the cell atlas in human [24] and mouse [25]. This study addresses the unmet demand for studying condition-specific gene regulatory network using scRNA-Seq data. We proposed and implemented a novel bioinformatics algorithm, scdNet, for systematic identification of gene pairs with regulatory strengths significantly changed between two groups of single cells. We adopted a sample-size correction transformation on correlation coefficients to cope with the sparsity of scRNA-Seq data. Using simulated datasets, we demonstrated the tolerance of scdNet to gene-gene covariances, relative power of noises, and number of single cells, as well as its great computational efficiency. We also applied the method to study two real-world datasets. CTC is a minimally invasive liquid biopsy strongly indicated for investigation of metastasis [26], prediction of treatment response [12, 13], and risk assessment of cancers [27, 28]. Our work is a unique extension into the underlying mechanisms of CTCs in treatment response of metastatic prostate cancer. By comparing CTCs obtained from patients responsive and naïve to a second-generation anti-androgen therapy, we constructed an intertwined gene regulatory network. Our data are in line with previous in vitro studies in the critical role of mitochondria and oxidative stress in the development of hormone-refractory prostate cancer [29–31]. In a non-cancer setting, scdNet also identified biologically meaningful results related to early development of mouse embryos. Overall, we demonstrated the feasibility of our method using simulated and real datasets. We expect the method to be widely applicable to different studies of biomedicine with the emerging applications of scRNA-Seq.

## Conclusions

Here we present a novel bioinformatics tool, namely scdNet, for a fast and comprehensive inference of differential gene regulatory networks out of scRNA-Seq data. Performance and computation efficiency of scdNet were demonstrated by simulation analysis. Applying the tool to a dataset of prostate cancer, we showed the involvement of mitochondria-related biological functions in anti-androgen resistance. We also illuminated crucial biological functions regulating early development of mouse embryos. Taken together, our data suggest wide applications of scdNet in exploring differential networks out of the rapidly increasing scRNA-Seq studies.

## Abbreviations

CTC: Circulating tumor cell
GO: Gene Ontology
scdNet: scRNA-Seq-based differential network analysis
scRNA-Seq: Single-cell RNA sequencing
